# A bird-eye view of diaphragmatic endometriosis: current practices and future perspectives

**DOI:** 10.3389/fmed.2024.1505399

**Published:** 2024-11-15

**Authors:** Antoine Naem, Horace Roman, Dan C. Martin, Harald Krentel

**Affiliations:** ^1^Department of Obstetrics, Gynecology, Gynecologic Oncology and Senology, Bethesda Hospital Duisburg, Duisburg, Germany; ^2^Faculty of Mathematics and Computer Science, University of Bremen, Bremen, Germany; ^3^Franco-European Multidisciplinary Endometriosis Institute, Clinique Tivoli-Ducos, Bordeaux, France; ^4^Franco-European Multidisciplinary Endometriosis Institute Middle East Clinic, Burjeel Medical City, Abu Dhabi, United Arab Emirates; ^5^Department of Gynecology and Obstetrics, Aarhus University Hospital, Aarhus, Denmark; ^6^Department of Obstetrics and Gynecology, University of Tennessee Health Science Center, Memphis, TN, United States

**Keywords:** endometriosis, diaphragm, shoulder pain, excision, ablation, progression

## Abstract

Diaphragmatic endometriosis is one of the most common localization of extra-pelvic endometriosis and may cause debilitating symptoms such as cyclic shoulder pain, right upper abdominal pain, and right-sided chest pain. Diaphragmatic endometriosis may also be asymptomatic. The exact mechanisms by which diaphragmatic endometriosis originates are unknown. The high correlation between severe pelvic endometriosis and diaphragmatic endometriosis suggests that the latter originates from endometriotic cells that reach the upper abdomen by circulating with the peritoneal fluid current. Robust evidence regarding the preoperative diagnosis and optimal management of diaphragmatic endometriosis is lacking. Most reports rely on Magnetic Resonance Imaging (MRI) for the radiologic diagnosis of diaphragmatic endometriosis. Although its sensitivity ranged between 78% and 83%, MRI was found to underestimate the extent of diaphragmatic endometriosis in comparison with the surgical findings. Accumulating evidence indicates that asymptomatic diaphragmatic endometriosis is very unlikely to progress, and therefore, could be left *in situ* when incidentally found. The efficiency of ablative and excisional approaches for symptomatic endometriosis has not been assessed thoroughly to date. In addition, it is unclear whether combining the laparoscopic approach with video-assisted thoracoscopy (VATS) may result in an optimized result. This gap exists due to the lack of data about the association between diaphragmatic and thoracic endometriosis. In this review, we aimed to provide a state of the art description of the current knowledge and gaps about the pathogenesis, diagnostics, and treatment modalities of diaphragmatic endometriosis.

## 1 Introduction

Endometriosis is often a complex, chronic inflammatory disease that is diagnosed in nearly 10% of reproductive-age women (1, 2). Current estimates suggest that the overall number of diagnosed endometriosis patients is up to 190 million women worldwide (1, 2). Endometriosis is classically defined by the presence of endometrium-like glands and/or stroma out of the uterus. Although this definition has been widely adopted, recent views indicate that symptomatic endometriosis is a systemic multifactorial disease with predisposing genetic and epigenetic dysregulations (3, 4). For instance, patients with endometriosis were reported to have defective peritoneal clearance of the hemoglobin metabolites, leading to increased oxidative stress in the pelvic cavity (5). Moreover, elevated cytokines concentrations and abnormal leukocyte activities were found in the pelvic milieu of the affected patients (6). Similarly, endometriosis patients were reported to have higher serum levels of IL-1β, IL-6, and TNF-*α* than healthy controls (7). The eutopic endometria of patients with endometriosis have altered differentiation processes and subsequently, an abnormal cellular spatial distribution (8). Patients with endometriosis were also reported to have increased uterine peristalsis, impaired folliculogenesis, and neural alterations in the peripheral and central nervous system (9–11). Therefore, the aberrantly located endometriotic implants and nodules could be considered the morphologic manifestations of highly complex and interwoven mechanisms that lead eventually to a variety of symptoms and organ malfunction. It is noteworthy that the aforementioned dysregulations may not be present in the entire population of endometriosis patients. Heterogeneities in the disease etiologic mechanisms probably exist; this is mainly reflected by the diversity of the endometriosis forms, localizations, symptomatology, and responsiveness to the available treatments (12). Such variations make a single theory explanation of the pathogenesis of endometriosis unlikely, and on the contrary, support the suggestion that endometriosis has several subtypes with different trajectories determined by distinctive genetic and epigenetic profiles (3, 4).

Peritoneal implants and deep nodules of endometriosis take predominantly intrapelvic localizations, with the ovaries and uterosacral ligaments being the most common sites of pelvic endometriosis (13, 14). Nonetheless, endometriosis may be encountered in extrapelvic localizations in 9–15% of patients (15, 16). Despite its rarity, diaphragmatic endometriosis and abdominal wall endometriosis could be considered the most common forms of extrapelvic endometriosis (17).

The prevalence of diaphragmatic endometriosis ranges between 0.67% and 4.7% (18, 19). Most diaphragmatic lesions are superficial and limited to the peritoneum, while endometriotic nodules infiltrating the muscular layer of the diaphragm are less common (20). As with all endometriotic lesions, diaphragmatic endometriosis may be symptomatic or asymptomatic. When symptomatic, cyclic shoulder pain, right upper abdominal pain, and right-sided chest pain are its typical symptoms (21).

**Figure 1:**
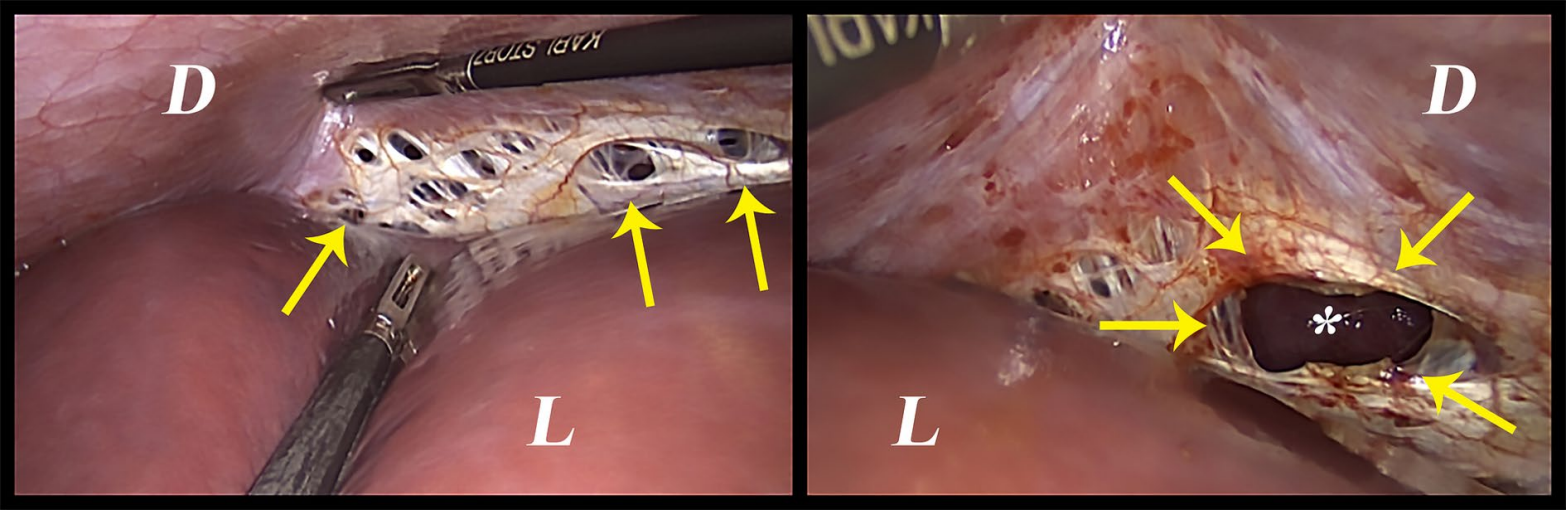
Intraoperative image of spontaneous diaphragmatic perforations that are thought to be related to diaphragmatic endometriosis. D: Diaphragm, L: Liver, Arrows: the perforations, *: the lung.

On the other hand, the thoracic endometriosis syndrome—characterized by catamenial pneumothorax, catamenial hemothorax, catamenial hemoptysis, and pulmonary nodules—is attributed to intrathoracic endometriosis affecting the parietal and visceral pleural surfaces, and less frequently, the pulmonary parenchyma (22). Nonetheless, it is important to note that catamenial pneumothorax *per-se* may not always refer to the presence of intrathoracic endometriosis. Although the pathophysiology is unclear, catamenial pneumothorax could be attributed to the trans-diaphragmatic passage of air from the genital tract (23). This happens due to congenital or endometriosis-associated perforations in the diaphragm (Figure 1). Air could leak from outside the body to the peritoneal cavity during menstruation due to the liquefaction of the cervical mucosal plug. The entry of the air could be induced by physical activities, sexual intercourse, or the abnormal uterine contractions (23, 24). The observation of pneumoperitoneum and pneumothorax is rare, but it was reported in the literature (24). Therefore, one should be aware of all possibilities and keep all the suggested mechanisms of this entity in mind before assuming any diagnosis.

It should be noted that diaphragmatic and thoracic endometriosis were always treated as two separate entities in the available literature. To date, there are few estimates about the coexistence of diaphragmatic and thoracic endometriosis. Individual case reports and case series confirm this possibility but remain incapable of providing definitive conclusions (25–27). More recently, Ancona et al. (28) reported the coexistence of diaphragmatic and thoracic endometriosis in 2.2% of their sample. It should be noted that this estimate was based on the abdominal trans-diaphragmatic examination of the thoracic cavity (28).

We aim through this work to discuss the available pieces of evidence regarding the pathogenesis of diaphragmatic endometriosis and its possible relations to deep pelvic and thoracic endometriosis. We also aim to provide a constructive appraisal of the available data regarding the surgical therapy of diaphragmatic endometriosis, its expected benefits, and long-term effectiveness.

## 2 The pathogenesis of diaphragmatic endometriosis

The retrograde menstruation peritoneal hypothesis by Sampson has been widely implicated in describing the origin of the different forms of endometriosis. Briefly, this hypothesis attributes peritoneal endometriosis to the implantation of regurgitated endometrial fragments that reach the peritoneal cavity through the Fallopian tubes during menstruation (29). Like all cell origin theories, the retrograde menstruation hypothesis is problematic because, it merely explains the origin of peritoneal endometriotic lesions. This is mainly because the retrograde menstruation theory and some other cell-of-origin theories do not explain deep endometriosis, ovarian endometriomas, pulmonary and more distal endometriosis. Moreover, retrograde menstruation occurs in 90% of women (30), while endometriosis prevails in about 10% only (1). Therefore, a pathogenic mechanism to explain the window-of-opportunity, method of activation, and determinates of growth, stabilization, or regression of endometriosis is needed.

Since the origin of endometriosis is still debatable and unclear, the pathogenesis of diaphragmatic endometriosis—as a subtype of the disease—will carry intuitively the same arguments. The retrograde menstruation hypothesis was also implicated in the pathogenesis of diaphragmatic endometriosis (31). The regurgitated endometrial fragments were suggested to reach the upper abdomen through the right paracolic gutter by circulating with the physiologic peritoneal fluid current (31). After reaching the right hypochondrium, the current is disrupted by the falciform ligament, which limits the access of the circulating endometrial cells to the left hypochondrium. The right-sided predominance of diaphragmatic endometriosis and its tendency to take superficial forms are arguments in favor of this hypothesis (20, 21).

Nonetheless, it is quite unclear whether the circulating cells originate from the eutopic endometrium (31) or from the dissemination of endometriotic cells originating from coexisting pelvic endometriosis (23). It is established that diaphragmatic endometriosis coexists almost always with superficial or deep pelvic endometriosis. Moreover, several reports indicate that the presence of diaphragmatic endometriosis often reflects the coexistence of more advanced stages of the disease in the pelvis (18, 20, 21, 32). This postulation gains further strength from the fact that the endometriotic epithelial and stromal cells showed clonal expansion ability *in vitro* (33). More recently, mutational profiling of different endometriotic lesions taken from the same individuals demonstrated that lesions at different anatomical locations belong to the same clone, and thus, share the same origin (34). When considering the high association of diaphragmatic endometriosis with pelvic endometriosis, it is quite plausible to think that pelvic endometriosis gives rise to diaphragmatic endometriosis by means of clonal expansion and dissemination. However, the actual origin of those lesions –whether it is the eutopic endometrium, endometriosis, bone morrow stem cells, or a different source- remains unknown.

Another question worth asking is whether thoracic pleural endometriosis originates through the same mechanisms of diaphragmatic endometriosis, and thus, diaphragmatic endometriosis could be considered “the precursor” of thoracic endometriosis.

It is noteworthy that around half of patients with diaphragmatic endometriosis suffer from infertility according to multiple studies (18, 20, 21). Therefore, diaphragmatic endometriosis, infertility, and deep pelvic endometriosis may be the characteristics of a unique but severe subtype of endometriosis. Those postulations are subject to selection and other biases, and need confirmation by future genetic and epigenetic studies.

## 3 The treatment of diaphragmatic endometriosis

The available management approaches for symptomatic endometriosis could be generally categorized into medical and surgical therapies. While surgical excision or ablation of endometriosis are cytoreductive techniques, the current medical therapies are either analgesic, anti-inflammatory, or hormonal suppressive medications (12). Growing evidence indicates that hormonal suppression of endometriosis results in decreasing the deep nodule size and the lesions’ activity (35–37). More recently, Kalaitzopoulos et al. (37) demonstrated that administering hormonal therapies for at least 3 months results in decreasing the endometriotic implants’ size and vascularization, with a remarkable anti-inflammatory effect. On this basis, the available medical approaches are prone to relieve the symptoms of the disease through decreasing the endometriosis activity or progression, without eliminating endometriosis or treating its underlying etiologies.

Surgery, on the other hand, is often indicated when patients are intolerant to the side effects of hormonal treatments, when the medical therapies fail to relieve the symptoms, when patients are seeking fertility, or when organ dysfunction is suspected. Although surgery is deemed beneficial in cases of deep endometriosis infiltrating the pelvic organs and neural structures (38, 39), it is not quite clear whether excising or ablating superficial lesions is effective in treating pain or infertility (40, 41). Furthermore, incomplete excision of endometriosis is accused of higher postoperative recurrence rates and symptom persistence (12). However, detecting all endometriotic lesions may be impossible. Thus, excising all endometriotic lesions may be impossible too and sometimes impractical as it may carry a higher risk of intra- and postoperative complications. In addition, accumulating data indicates that most endometriosis lesions tend to stabilize or regress over time (42–44), which means that inactive or fibrotic lesions and nodules could be left *in situ*, when asymptomatic (45).

The aforementioned therapeutic dilemmas exist in the management strategies of diaphragmatic endometriosis, but due to its rarity, the available studies focusing on it are very few (18, 20, 21, 32, 46, 47). Although the medical treatments were reported to be the first-line treatment for symptomatic diaphragmatic endometriosis (48), we found no studies assessing the effectiveness of analgesics and hormonal medications in controlling the symptoms of diaphragmatic endometriosis. Therefore, administering those medications to treat diaphragmatic endometriosis should be empirical with close patient follow-up to monitor their responsiveness to therapy. A standardized approach to diaphragmatic endometriosis was suggested in the report of Roman et al. (49). The authors recommended placing the patient in the left lateral decubitus position and excising the superficial lesions before performing a full-thickness resection of the diaphragm (49). The same group favored the robotic-assisted resection of big endometriotic lesions with partial or complete muscle infiltration, while the laparoscopic approach with plasma energy ablation is preferred for smaller superficial lesion with limited extension (50).

Currently, the available studies focus mainly on the surgical approach to diaphragmatic endometriosis. Most of these studies reported the used techniques and the complication rates, but the majority were not powered enough to determine the long-term outcomes of diaphragmatic endometriosis surgery. The follow-up as done in such studies was always limited to either short follow-up periods or the lack of structured and standardized postoperative pain assessment. Another concern about surgical interventions on the diaphragm is the possible onset of new symptoms and/or development of chronic post-surgical pain, as previously reported (21).

The lack of standardization of the operation technique is another drawback of diaphragmatic endometriosis surgery. It is well-known that the major part of the right hemidiaphragm is hidden behind the right hepatic lobe, and thus, liver mobilization may be necessary in most cases to guarantee a better detection of endometriosis. Redwine (19) stressed that sentinel lesions of the anterior parts of the right hemidiaphragm often indicate the presence of a more severe disease hidden behind the liver (19). Furthermore, the preferred route of surgery –abdominal, thoracic, or a combination of both- is yet to be determined. In the study of Nezhat et al. (26), the thoracic and visceral sides of the diaphragm were involved in 76% of patients, while the sole involvement of the visceral side was reported in only 8% of patients (26). It is noteworthy that in the same study thoracic endometriosis was found in 16% of cases (26). Therefore, a combination of laparoscopy and video-assisted thoracoscopy could be necessary in a considerable proportion of symptomatic patients to ensure a better detection and elimination of the disease. Some surgeons also prefer to do video-assisted thoracoscopy in all patients with diaphragmatic endometriosis to detect intrathoracic lesions that may be missed during trans-diaphragmatic exploration of the thoracic cavity (51). A thoracic approach to diaphragmatic endometriosis was also recommended by Roman et al. (50) for endometriotic lesions infiltrating the central tendon of the diaphragm to allow a better recognition and isolation of the phrenic nerve and preventing its injury. We would also recommend a careful examination of the visceral pleura since most pulmonary endometriotic lesions were reported to take interlobular localization (52).

Lastly, it should be noted that the likelihood of asymptomatic lesions to progress or become symptomatic over time is quite low and the lesions could be left *in situ*. In the study of Naem et al. (21), all four patients who were asymptomatic remained asymptomatic, compared to two out of three patients in the study of Nezhat et al. (53).

## 4 Discussion

Diaphragmatic endometriosis is a vague variant of endometriosis with very little known regarding its pathogenesis, diagnosis, and treatment. The typical symptoms of diaphragmatic endometriosis and thoracic endometriosis should be considered in every patient with pelvic endometriosis. The diagnosis of diaphragmatic endometriosis is presumed at the interrogatory and mostly done intraoperatively as a finding in the context of laparoscopy for pelvic endometriosis. Magnetic resonance imaging (MRI) is the most reported imaging modality for the preoperative diagnosis of diaphragmatic endometriosis. To the best of our knowledge, there is one study available that assessed its sensitivity and the reproducibility of its results in patients with diaphragmatic endometriosis (54). Although the MRI sensitivity ranged between 78% and 83%, the authors indicated that the examiners often underestimated the extent of the disease when diaphragmatic endometriosis is diagnosed radiologically. In addition, it should be noted that the study by Rousset et al. (54) included patients with histologically-proven diaphragmatic endometriosis or patients with thoracic symptoms responsive to hormonal therapy. Therefore, the generalizability of the results is limited. In another series, the sensitivity of the MRI is reported to be 48.3% (28).

In our opinion, there are two crucial messages to be delivered by this study. Firstly, when an MRI is positive for endometriosis, the surgeons should keep in mind that the extent of the disease is beyond the radiologic findings. Secondly, MRI could be better done during menses, since the authors reported the detection of an endometriotic lesion in Morison’s pouch that was not observed in a preceding MRI that was performed out of the menstruation period. Endometriotic lesions of the diaphragm could manifest as high-signal intensity in the fat-suppressed T1 weighted imaging. Depending on the lesion’s size, the endometriotic lesions could be classified radiologically into micronodules (<5 mm), nodules (≤3 cm), and plaques (≥3 cm) (54). Another indirect sign of diaphragmatic endometriosis is the “Air-Filled Bubbles” as observed by Quercia et al. (55) in the coronal section of the computed tomography scan.

When surgery is indicated, the optimal surgical route and patient positioning remain a matter of debate. In their recent review, Ceccaroni et al. (56) recommended tailoring the approach to superficial diaphragmatic endometriosis based on the lesions’ characteristics and extension. The approaches could range from bipolar coagulation to diaphragmatic peritonectomy (56). The choice of how to deal with superficial diaphragmatic endometriosis depends on the operator’s experience and availability of the surgical equipment. We believe that ablation or excision are equally effective in superficial diaphragmatic endometriosis, when complete destruction of the lesion is achieved.

In the series of Ancona et al. (28), transabdominal robotic-assisted resection of the diaphragmatic lesions was reported to be effective. After a 3-month follow-up, the symptoms’ relief rate was 80%. The authors believe that the increased range of motion—especially in a narrow surgical field—and the improved visualization of the diaphragmatic surface due to the better 3D image of the robotic platform helped detecting all diaphragmatic lesions and facilitated a more precise and radical excision of diaphragmatic endometriosis, as well as an easier suturing of the diaphragmatic defects (53). The safety profile of this procedure is also deemed acceptable with the intraoperative complication rate and the long-term postoperative complication rate being 1.7 and 6.6%, respectively. The intraoperative complication was a hepatic injury leading to diffuse hemorrhage and a conversion to laparotomy; it occurred in one patient only. While the long-term complications were three cases of liver herniation and one case of pneumothorax (28).

Finally, the patient positioning is an important point to consider when dealing with diaphragmatic endometriosis. The left lateral decubitus with a 10° anti-Trendelenburg position could be the “position of choice” when carrying out diaphragmatic surgery alone. This position improves the visualization of the posterior part of the right diaphragmatic dome and helps avoiding the mobilization of the liver. This in turn improves the access to and resection of all the visualized diaphragmatic lesions (28, 51). However, the dorsal lithotomy may still be needed in cases of bilateral involvement of the diaphragmatic domes and in cases where concomitant pelvic and diaphragmatic endometriosis surgery should be performed (51).

## 5 Conclusion

Diaphragmatic endometriosis has a vague pathogenesis but current studies suggest a strong association with pelvic endometriosis and infertility. Therefore, the management of diaphragmatic endometriosis should be decided while taking into consideration the symptoms severity, the level of severity of pelvic endometriosis, and the patient’s willingness to conceive. When surgery is decided, the extent and radicality of the surgical approach should be tailored to the patient’s condition and symptomatology. Due to a lack of robust evidence, a combined thoracic-abdominal minimally invasive approach should be considered, but solid recommendations cannot be made. Although excision is the preferred surgical approach, ablation could be considered for superficial peritoneal lesions without partial- or full-thickness muscular infiltration. Asymptomatic lesions are more likely to remain stable than become symptomatic. Therefore, those could be managed expectantly. Future research should focus on the long-term outcomes of diaphragmatic endometriosis surgery, the superiority or non-inferiority of ablative and excisional techniques, and most importantly, the association between thoracic and diaphragmatic endometriosis to decide on the necessity of a combined thoracic-abdominal surgical route.
